# The *Juan *non-LTR retrotransposon in mosquitoes: genomic impact, vertical transmission and indications of recent and widespread activity

**DOI:** 10.1186/1471-2148-7-112

**Published:** 2007-07-09

**Authors:** James K Biedler, Zhijian Tu

**Affiliations:** 1 Department of Biochemistry, Virginia Polytechnic Institute and State University, Blacksburg, VA 24061, USA

## Abstract

**Background:**

In contrast to DNA-mediated transposable elements (TEs), retrotransposons, particularly non-long terminal repeat retrotransposons (non-LTRs), are generally considered to have a much lower propensity towards horizontal transfer. Detailed studies on site-specific non-LTR families have demonstrated strict vertical transmission. More studies are needed with non-site-specific non-LTR families to determine whether strict vertical transmission is a phenomenon related to site specificity or a more general characteristic of all non-LTRs. *Juan *is a Jockey clade non-LTR retrotransposon first discovered in mosquitoes that is widely distributed in the mosquito family *Culicidae*. Being a non-site specific non-LTR, *Juan *offers an opportunity to further investigate the hypothesis that non-LTRs are genomic elements that are primarily vertically transmitted.

**Results:**

Systematic analysis of the ~1.3 Gbp *Aedes aegypti *(*Ae. aegypti*) genome sequence suggests that *Juan-A *is the only *Juan*-type non-LTR in *Aedes aegypti*. *Juan-A *is highly reiterated and comprises approximately 3% of the genome. Using minimum cutoffs of 90% length and 70% nucleotide (nt) identity, 663 copies were found by BLAST using the published *Juan-A *sequence as the query. All 663 copies are at least 95% identical to *Juan-A*, while 378 of these copies are 99% identical to *Juan-A*, indicating that the *Juan-A *family has been transposing recently in evolutionary history. Using the 0.34 Kb 5' UTR as the query, over 2000 copies were identified that may contain internal promoters, leading to questions on the genomic impact of *Juan-A*. *Juan *sequences were obtained by PCR, library screening, and database searches for 18 mosquito species of six genera including *Aedes*, *Ochlerotatus*, *Psorophora*, *Culex*, *Deinocerites*, and *Wyeomyia*. Comparison of host and *Juan *phylogenies shows overall congruence with few exceptions.

**Conclusion:**

*Juan-A *is a major genomic component in *Ae. aegypti *and it has been retrotransposing recently in evolutionary history. There are also indications that *Juan *has been recently active in a wide range of mosquito species. Furthermore, our research demonstrates that a Jockey clade non-LTR without target site-specificity has been sustained by vertical transmission in the mosquito family. These results strengthen the argument that non-LTRs tend to be genomic elements capable of persistence by vertical descent over a long evolutionary time.

## Background

TEs, or mobile genetic elements, are integral components of the eukaryotic genomes. Because they have the ability to replicate and spread in the genome as primarily "selfish" genetic units [1], TEs tend to occupy significant portions of the genome [2]. Recent evidence suggests that the "selfish" property may have enabled TEs to provide the genome with potent agents to generate tremendous genetic and genomic plasticity [3]. TEs transpose through either RNA-mediated or DNA-mediated mechanisms [4]. DNA-mediated TEs generally transpose by a cut-and-paste process, directly from DNA to DNA. RNA-mediated TEs transpose by a replicative process that involves transcription, reverse transcription, and integration of cDNA molecules. TEs in this category include the long terminal repeat (LTR) retrotransposons, non-LTRs, or long interspersed nuclear elements (LINEs), and short interspersed nuclear elements (SINEs).

It has been proposed in models of the lifecycle of DNA-mediated TEs [5-7] that most TEs will eventually become inactivated in a given species, which underscores the importance of horizontal transfer for TE survival, a mechanism that allows TEs to invade a naïve genome. Horizontal transfers DNA-mediated TEs are well documented [8-10]. There have also been cases of non-LTR horizontal transfer proposed [11-16], the most convincing case involving RTE clade elements [11,15,16]. RTE non-LTRs were first found in *C. elegans *and encode a single open-reading frame (ORF) containing reverse transcriptase and endonuclease activities [17]. In contrast, it has been argued that there is no reliable evidence of non-LTR horizontal transfer between eukaryotes in the last 600 million years according to age vs. divergence analysis [18,19]. Research involving arthropod R1 and R2 families, which are site-specific non-LTRs that insert into 28S ribosomal RNA genes, shows vertical inheritance of these elements since the origin of the *Drosophila melanogaster *species subgroup, approximately 17–20 million years ago (MYA) [20]. Even multiple lineages have been found to coexist in the rRNA loci and be maintained by vertical descent [21]. Other studies on R1 and R2 lineages concluded that they have been vertically transmitted since the inception of the *Drosophila *genus, approximately 60 MYA or longer [22,23]. The site-specificity of R1 and R2 may result in a bias toward vertical transmission as site-specificity could offer a "safe haven", protecting the genome from deleterious insertions elsewhere.

*Juan-A*, a Jockey clade non site-specific non-LTR from *Ae. aegypti *has been reportedly involved in potential horizontal transfer between the non-sibling species *Ae. albopictus *and *Ae. polynesiensis *[24]. However, Crainey and colleagues [25] recently suggest that vertical transmission explains the evolutionary relationship between *Juan *elements in *Ae. aegypti, Ae. albopictus*, and *Culex pipiens quinquefasciatus *(herein referred to as *C. quinquefasciatus*). They also did not find evidence to support horizontal transfer of CR1 clade elements *Q *and *T1 *in mosquitoes, although an earlier report suggested horizontal transfer could conceivably explain the identities and distributions of CR1 families in diverse taxa [26]. Here we report a detailed evolutionary study of *Juan *in the mosquito family *Culicidae*. Sequences of full-length *Juan *elements have been reported from the yellow fever mosquito *Ae. aegypti *[24] and the house mosquito *C. quinquefasciatus *[27]. In this study, we have obtained sequences of *Juan *elements from 18 mosquito species of six genera. Our results support that non-LTRs are able to sustain their activity over long periods of evolutionary time relying primarily on vertical transmission while not excluding the possibility of rare horizontal transfer. Our whole-genome analysis suggests that *Juan-A *has been retrotransposing recently in evolutionary history, and it occupies approximately 3% of the *Ae. aegypti *genome. We have also discussed the potential evolutionary impacts of *Juan-A *in the *Ae. aegypti *genome.

## Results

### *Juan *in *Aedes aegypti*: abundance and recent activity

**Figure 1:**
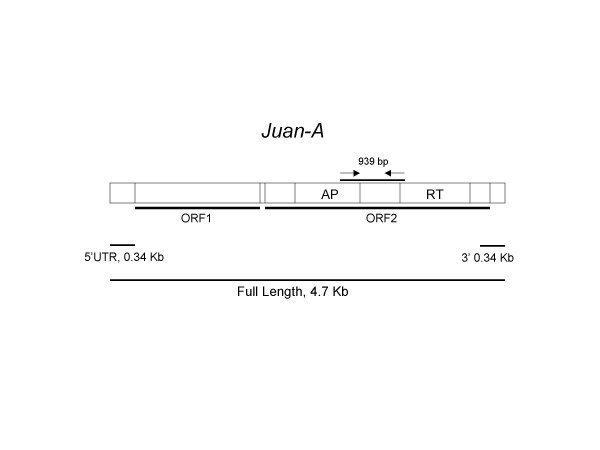
**Structural organization of the *Juan-A *element of *Aedes aegypti***. ORF1 encodes a nucleic acid binding protein and ORF2 encodes both an apurinic/apyrimidinic (AP) endonuclease and reverse transcriptase (RT) domain. Arrows indicate the 939 bp region amplified by PCR that was used for phylogenetic inference. A canonical polyadenylation signal sequence is present in the 3' end of *Juan-A* (not shown) Regions used for copy number determination by database search in Table 1 are shown by horizontal lines.

**Figure 2:**
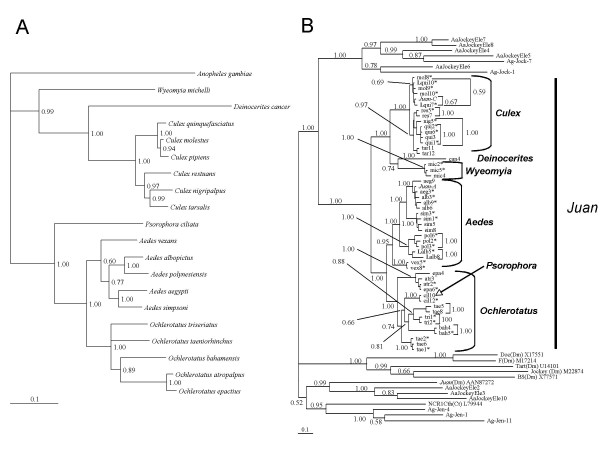
**Phylogenetic comparison of *Juan *sequences and their hosts**. A. Consensus tree of host phylogeny constructed with MrBayes (see methods) using nucleotide sequences of Vg-C, a single copy gene. Vg-C from *An. gambiae *is used to root the tree. Clade credibility values from 150,000 generations are given at each node. B. Consensus tree constructed with MrBayes using conceptually translated sequences of *Juan *from PCR and genomic database (Aa, *Ae. aegypti*, Ag, *An. gambiae*). Clade credibility values from 200,000 generations are given at each node or beside brackets. Ag-Jen-4 and other *An. gambiae *sequences correspond to families previously identified (Biedler and Tu 2003). Jockey elements from *D. melanogaster *(Dm) and *C. tentans *(Ct) are used to root the tree. Accessions are given beside sequence names. An asterisk indicates that the reading frame was intact. A bold capital "L" indicates that the sequence was obtained from a genomic library. The first three letters of a species name is used to label PCR and library sequences. Genus names are in bold beside brackets. *Juan-A *is from *Ae. aegypti *and *Juan-C *is from *C. pipiens*. Abbreviations: *Ae. aegypti *(*aeg*); *Ae. albopictus *(*alb*); *Ae. simpsoni *(*sim*); *Ae. polynesiensis *(*pol*); *Ae. vexans *(*vex*); *C. molestus *(*mol*); *C. quinquefasciatus *(*qui*); *C. restuans *(*res*); *C. tarsalis *(*tar*); *D. cancer *(*can*); *O. atropalpus *(*atr*); *O. bahamensis *(*bah*); *O. epactius *(*epa*); *O. taeniorhinchus *(*tae*); *O. triseriatus *(*tri*); *P. ciliata *(*cil*); *W. michelli *(*mic*). Number indicates clone from PCR. *Ae. aegypti *and *An. gambiae *sequences from genomic database (e.g. Ag-Jock-1, AaJockeyEle2) can be found in the TEfam database [57]. See additional files 1 and 2 for alignments used for phylogenetic inference.

*Juan-A *contributes significantly to the genome size of *Ae. aegypti*, determined to be approximately 3% by RepeatMasker (see Methods). A highly variable copy number is found depending on what query region and identity criteria are used (Figure 1, Table 1). *Juan-A *appears to be the only *Juan*-type element in *Ae. aegypti*. After masking the genome sequence for *Juan-A *(80% nt identity) with RepeatMasker [28], tBLASTn [29] with *Juan-A *amino acid (aa) sequence was used to identify closely related families. The two closest families found, AaJockeyEle4 and AaJockeyEle6 have approximately 37% aa identity to *Juan-A *in the same region used for phylogenetic inference (Figure 1, Figure 2B). AaJockeyEle4 and AaJockeyEle6 are part of divergent Jockey clade families, not *Juan*-type elements (Figure 2B).

**Table 1:** Copy numbers of *Juan-A *in *Ae. aegypti *determined by genomic analysis.

	**Number of copies with greater nt identity than indicated to the *Juan-A *query**					
***Juan-A *sequence region**	**99%**	**98%**	**97%**	**95%**	**80%**	**70%**
Full-length^a^	378	637	662	663	663	663
5' UTR 0.34 Kb	957	1596	1920	2137	2274	2274
3' 0.34 Kb	180	867	1302	2886	4852	4853

^a ^includes sequences having 90% length compared to a full-length *Juan-A *(see methods)

Analysis of *Juan-A *in *Ae. aegypti *reveals that the family has undergone recent amplification, evidenced by a high degree of homogeneity between copies (Table 1). We have chosen to look at groups of sequences having various identities to the *Juan-A *query to obtain a more comprehensive picture of *Juan *evolution in the genome. Using lower identity criteria should allow the identification of both older retrotransposed copies as well as copies from more divergent *Juan-A*-related sequences. There are 663 copies of full-length or nearly full-length *Juan-A*, defined as having at least 90% length and at least 70% nucleotide identity compared to the 4.7 kb published *Juan-A *sequence. All 663 copies are at least 95% identical to *Juan-A*, suggesting that there are no divergent subgroups among these nearly full-length *Juan *elements. Three hundred seventy-eight of the 663 copies are 99% identical to *Juan-A*, indicating that the *Juan-A *family has been transposing recently in evolutionary history. There is no appreciable difference in copy numbers between using 80% and 70% nt identity cutoffs (Table 1). As stated in the Methods, many gaps exist (36206 contigs) in the *Ae. aegypti *genome sequence assembly. We did not determine how many *Juan-A *copies were truncated by these gaps so it is possible that many more full-length copies exist in the genome. In addition, we used a gap parameter value of 50 bp for our BLAST processing program. Therefore, *Juan *sequences with insertions of over 50 bp are not counted as full-length sequences.

We also used 5' and 3' end regions as queries to get a general impression of *Juan *representation and activity in the genome. A higher number of 5' ends were found than 3' ends when looking at those copies that had greater than 97% identity to the query, a curious result. Using BLAST through NCBI, many *Juan-A *hits were found to EST sequences from full-length cDNA libraries (not shown). Several hits to the 3' end of *Juan-A *were from sequencing reactions using oligo dT primers, indicating that these are from polyadenylated transcripts.

### *Juan *is widely distributed in Culicinae

*Juan *family sequences were obtained by PCR from 18 species of six genera including *Aedes*, *Ochlerotatus*, *Psorophora*, *Culex*, *Deinocerites*, and *Wyeomyia *(Table 2). PCR products from each species were cloned and sequenced. Additional sequences were obtained from *Ae. albopictus *and *C. quinquefasciatus *genomic libraries. PCR with 12 other species either yielded no product, or bands of the expected size that corresponded to other retrotransposons. These 12 species are: *Anopheles *(*An*.)*gambiae, An. stephensi, An. freenborni, An. quadrimaculatus, An. albimanus, Armigeres subalbatus, Culex erraticus, Culiseta melanura, Mansonia dyari, Mansonia titillans, Psorophora ferox*, and *Toxorhynchites amboinensis*. Most but not all of the *Juan *"negative" species were distantly related to the *Juan *"positive" species. Failure of PCR amplification could result from mutations in the primer target sequences or the absence of a "true" *Juan *in the species. No PCR products were obtained from any Anopheline species. When *Juan-A *aa sequence is used for BLAST vs. the *An. gambiae *genome sequence, the most significant hit retrieved is Ag-Jen-4 (Figure 2B) having only ~36% amino acid identity in the same region used for PCR. Additional sequences added from the *Ae. aegypti *and *An. gambiae *genome show the presence of several divergent Jockey families that are paralogous to *Juan *(Figure 2B).

**Table 2:** Species from which *Juan *sequences were obtained by PCR or library screening.

**Genus**	**Species**
*Aedes*	aegypti, albopictus*, polynesiensis, simpsoni, vexans
*Ochlerotatus*	*atropalpus, bahamensis, epactius, taeniorhinchus, triseriatus*
*Psorophora*	*ciliata*
*Culex*	molestus, nigripalpus, quinquefasciatus*, restuans, tarsalis
*Deinocerites*	*cancer*
*Wyeomyia*	*michelli*

Note: An asterisk indicates that sequences were obtained by both PCR and library screening.

### *Juan *appears to have been active throughout the mosquito family

Six species have 3 or more *Juan *sequences that share a high degree of intragenomic nt identity (Table 3). Values shown in Table 3 are the mean of comparisons of each sequence vs. the consensus generated from that group of sequences. Sequence identity of PCR clones ranges from approximately 97.1% in *Ae. aegypti *to 99.4% at the nucleotide level in *C. quinquefasciatus*. Four sequences from *C. quinquefasciatus *have over 99% identity. These do not appear to come from the same copy of *Juan *in the genome since a deletion is present in one sequence and substitutions can be found at various positions among the different sequences. PCR and library clones from 16 of 18 species yielded sequences that do not have frameshifts or stop codons within this analyzed portion of the ORF (Figure 2B). Altogether, these results indicate recent activity of *Juan *in both closely related and divergent species.

**Table 3:** *Juan *sequences from several species of four genera have a high degree of sequence identity.

**Species**	**Nucleotide Identity**	**# Sequences compared**
*Ae. aegypti*	99.0%	768
*Ae. simpsoni*	97.1 +/- 0.3%	4
*C. molestus*	98.5 +/- 0.2%	3
*C. quinquefasciatus*	99.4 +/- 0.2%	4
*O. taeniorhinchus*	99.1 +/- 0.1%	3
*W. michelli*	97.5 +/- 0.7%	3

Note: only sequences in the same lineage are compared

### Negative selection has been acting on *Juan*

The rates of synonymous (*dS*) and nonsynonymous (*dN*) codon substitution have been commonly used as a measure of selection pressure. A value of *dS*/*dN *close to 1 is taken to indicate neutral selection as would be expected for a pseudogene. Values below and above 1 indicate positive and negative selection. Vitellogenin-C (Vg-C), a single copy yolk protein-encoding gene [30] was used as a comparison to *Juan*, because we had Vg-C sequences available for many species. It should be noted that Vg-C is known to be a relatively fast-evolving gene [30], but the fact does not affect the interpretation of *Juan dS*/*dN *values. The *dS*/*dN *ratio was calculated for all *Juan *sequences of the *Aedes/Ochlerotatus* and *Culex* genera that had intact reading frames. *Juan *sequences analyzed show a significant bias toward synonymous substitution, over 10 times that of the nonsynonymous rate. *dS/dN *values for *Juan *from the *Aedes/Ochlerotatus* and *Culex* genera were 10.7 (+/-2.9) and 12.3 (+/-2.3), respectively. Vg-C sequences from the *Aedes* genera had a value of 16.9 (+/-4.1). This is consistent with the interpretation that *Juan *has been retrotransposing in mosquito genomes, and this region is under negative selection due to functional constraint.

### Vertical transmission of *Juan *and a few cases of phylogenetic incongruence

Comparison of host phylogeny with TE phylogeny is one method used to address the question of vertical vs. horizontal transmission. A detailed mosquito phylogeny has been previously constructed using Vg-C [30]. We have only included Vg-C sequences from species for which *Juan *sequences were obtained in this study (Figure 2A). In addition, we have also obtained sequence for Vg-C from *Ae. simpsoni*, which was not available from the previous dataset [30]. We used nt sequences for phylogenetic inference as in the previous study, and our phylogeny is consistent with the phylogeny based on the larger Vg-C dataset [30].

Phylogenetic inference using Bayesian methods shows support for the vertical transmission of *Juan *in the mosquito family as comparison of *Juan *and host phylogenies shows overall congruence of tree topology with few exceptions (Figure 2A and 2B). *W. michelli *is basal to the *Culex *genus and *D. cancer *group in the Vg-C phylogeny (Figure 2A) while the *Juan *phylogeny (Figure 2B) shows *W. michelli *as a sister group to *D. cancer*. The *D. cancer *sequence is degenerate (note long branchlength) and therefore may complicate phylogenetic resolution here. Furthermore, *P. ciliata *is basal to the *Aedes *and *Ochlerotatus *genera in the host phylogeny. However, the *Juan *sequences isolated from *P. ciliata *are found within the *Ochlerotatus *genus. There are also indications of two sets of paralogous *Juan *sequences from *O. taeniorhinchus *(Figure 2B).

The *Juan *phylogeny suggests that horizontal transfer could have occurred in a few cases but the support is weak. One case involves *Ae. aegypti *and *Ae. albopictus*, in which 3 cloned PCR products from *Ae. albopictus *were nearly identical to sequences from *Ae. aegypti*. Sequences obtained by screening an *Ae. albopictus *genomic library are found grouped with *Ae. polynesiensis *sequences as expected according to known mosquito phylogeny. Another case involves *C. quinquefasciatus*, for which we also have sequences from both PCR and a genomic library. The two library sequences group with *C. molestus *and *C. pipiens *(*Juan-C*), as expected according to host phylogeny. However, the PCR sequences group most closely with *C. nigripalpus*. *O. atropalpus *(atr2, Figure 2B) and *O. epactius *(epa6, Figure 2B) sequences are almost identical with over 99% nucleotide identity, but they come from species that are in the same species complex where introgression may exist.

## Discussion

### Genomic impacts of *Juan-A *in *Ae. aegypti*

Juan contributes approximately 3% to the *Ae. aegypti *genome sequence while the entire TE complement is estimated to be 47% (*Ae. aegypti *genome consortium, unpublished). With its significant contribution to genome size and the presence of hundreds of highly homogeneous full-length or near full-length copies, a natural question concerns the genomic impact of *Juan*. TEs can cause chromosomal inversions by providing sites for ectopic homologous recombination and by other mechanisms [31]. It might be thought that the hundreds of highly homogeneous copies might contribute to genomic instability.

Most non-LTR families usually consist of a large majority of 5' truncated copies, which has been attributed to incomplete reverse transcription, template switching, or other mechanisms [32-35]. However, when using higher stringency for copy number determination (representing more recently amplified elements), there is a higher copy number of 5' ends of *Juan-A *sequences than 3' ends (Table 1). This could be a result of selection for 5' ends, selection against 3' ends, or possibly a distribution bias of 3' end insertion into regions that are underrepresented in the genome sequence. Full-length non-LTRs have been shown to contain their own self-sufficient internal pol II promoter in the 5'UTR [36-40]. It is interesting that so many 5'UTRs of *Juan-A *are present in the genome. These 5' UTRs, if functional as internal promoters, may produce a transcriptional burden. It is interesting to note that our reporter assays have not demonstrated promoter activity of the *Juan-A *5'UTR in cell lines from three mosquito species, while 5'UTRs of mosquito non-LTRs from 3 non-LTR clades have proven active in all 3 lines (not shown). Perhaps *Juan-A *is dependent on upstream promoter elements for transcription, as upstream sequences have been found to greatly influence the activity of the human L1 promoter activity [41]. Past analysis of *Juan*-C transcripts from cell culture showed that all transcripts analyzed were transcribed from upstream of the *Juan *element [42]. With its recent amplification and recent activity, the study of *Juan *may offer a good opportunity to increase our understanding the competing forces of non-LTR activity and host regulation.

### *Juan *evolution

To address the topic of vertical transmission and to analyze the distribution and evolution of *Juan *in Culicidae, a detailed phylogeny of the host species was needed. Most phylogenetic inferences of mosquitoes based on molecular data have been focused on the *Anopheles *genus due to its medical importance. More comprehensive analyses have been performed using the white gene [43] and Vg-C [30]. The Vg-C sequences available to us offered the most comprehensive phylogeny with many species from the *Aedes *and *Culex *genera, where *Juan *was discovered.

The Jockey clade is comprised of highly divergent families which have been found in several insect species [18,25,44,45]. Representatives of *Juan *have been reported in mosquitoes and in Drosophila [45]. However, those elements are distant relatives of the *Juan-A *and *Juan-C *elements (Figure 2B), which we are investigating in this study. We have focused on *Juan-A *and *Juan-C *(*Juan sensu stricto*) because use of paralogous sequences can lead to erroneous conclusions of phylogenetic relationships. Results from Crainey and colleagues (2005) are consistent with vertical transmission but they also included many paralogous sequences from three mosquito genera. As mentioned above, we focus on *Juan sensu stricto *and survey many mosquito species to investigate the question of *Juan *evolution. It is important to note that our results indicate that *JuanDm *[45] is not actually a Juan element, strictly speaking, since it groups with three divergent *Ae. aegypti *Jockey elements, having 99% support (Figure 2B). This underscores the importance of including many divergent representatives while performing phylogenetic inference.

Regarding the cases of potential horizontal transfer, there are alternative explanations. For the *Ae. albopictus *(alb 3, 6, 9, Figure 2B) and *O. epactius *(epa 6, Figure 2B) sequences, the first suspicion is genomic DNA contamination of the PCR reaction. The *Ae. albopictus *sequences obtained from a genomic library were found grouped with *Ae. polynesiensis*, as expected. It should be noted that the library was constructed from the Nepal strain and PCR was performed on the Oahu strain. Bensaadi-Merchermek, Salvado, and Mouches (1994) reported the absence of *Juan-A *from *Ae. albopictus *Oahu strain (1971). If our PCR results can be corroborated using other methods, this would suggest the horizontal transfer of *Juan-A *to this strain of *Ae. albopictus*. However, *Juan-A *was also reported absent from strains of *Ae. polynesiensis *and *O. triseriatus *[42], both species of which we were able to obtain PCR products that grouped phylogenetically as expected, supporting vertical transmission of these elements. For *C. quinquefasciatus*, sequences obtained from library screening correspond with the host phylogeny, being grouped in the *C. pipiens *species complex. In contrast, sequences obtained from PCR are found outside this group and placed closely with *C. nigripalpus *with approximately 94% nucleotide identity to nig5 (Figure 2B). Although possible, the nucleotide identities between the *C. quinquefasciatus *sequences and the *C. nigripalpus *sequence are not close enough to suspect genomic DNA contamination of the PCR. Another possibility is that different sublineages of *Juan *could have been sampled by PCR versus library screening. For example, there are two sublineages represented in *O. taeniorhinchus*. The amplification of *Juan *sequences from contaminating genomic DNA cannot be ruled out, especially when using degenerate primers with low stringency PCR conditions. This seems unlikely in the case of *C. quinquefasciatus*, because these multiple sequences form their own homogeneous group with high nucleotide identity. If they resulted from contaminating genomic DNA, then they would be expected to group with sequences of the contaminating species. In summary, there is evidence for multiple *Juan *lineages, which could explain some of the observed phylogenetic incongruence. However, further analysis is required to determine whether the phylogeny of the suspect sequences is due to horizontal transfer, genomic DNA contamination, or sampling of different sublineages.

## Conclusion

It has been proposed that the horizontal transfer of non-LTRs are rare events and few reported cases have strong supporting evidence without alternative explanations [18,19]. In contrast, there are many cases documented for the horizontal transfer of DNA-mediated TEs. Without excluding the possibility of horizontal transfers, we find that *Juan *family members do mirror their host's phylogeny closely, supporting the vertical transmission of these elements. Our results suggest the *Juan *family was able to sustain its activity in the mosquito family over long periods of evolutionary time. Estimates of the time since *Aedes *and *Culex *divergence would suggest that *Juan *has been maintained for at least 22–52 million years [46]. Furthermore, the presence of multiple Culicinae lineages approximately 120 MYA has been proposed [47], suggesting that Juan may have persisted for at least this time. Detailed studies involving the site-specific non-LTRs R1 and R2 in *Drosophila *showed that they are vertically transmitted and are maintained in their respective genomes [20,22,23]. It may be argued that vertical transmission of R1 and R2 over a long evolutionary time could be unique to site-specific non-LTRs. This study, which was performed in a different insect group using a non-LTR that does not exhibit site-specificity, strengthens the hypothesis that non-LTRs are able sustain their activity without the need of horizontal transfer. It will be interesting to see if other non-LTRs behave in a similar fashion, especially those from other clades and divergent taxa that have not been studied in detail.

## Methods

### PCR amplification of genomic DNA and cloning

Degenerate primers GDFNAKH (forward) and FKNMKAPG (reverse) (Sigma Genosys) were designed according to conserved amino acid sequence including 939 bp found in an alignment of ORF2 of the *Juan *element from *Juan-A *of *Ae. aegypti *and *Juan*-C of *C. pipiens *(Figure 1). In contrast to the commonly used RT region, we chose to use this less conserved region to increase resolution between sequences from closely related species. Genomic DNA was isolated from several individuals of a given species using the DNAzol Genomic DNA Isolation Reagent (Molecular Research Center). PCR was performed on genomic DNA from a total of 30 species of mosquitoes from 10 genera. The calculated Tms of the forward and reverse primers were 54.2°C and 62.7°C. Each 20 ul PCR reaction consisted of approximately 3 ng of genomic DNA, 1U of TakaRa Taq Polymerase (Takara), 1.5 mM MgCl_2_, and 0.2 mM each dNTP. PCR was performed by denaturation at 95°C for 90s and 30 cycles of 95°C for 30s, 48°C for 50s, and 72°C for 90s. Amplified products were size-separated on a 0.7% agarose gel and purified using the Sephaglass BandPrep Kit (Amersham Pharmacia Biotech). These products were cloned into the pCR 2.1 TOPO vector using the TOPO TA Cloning Kit version K2 (Invitrogen) or the pGEM-T Easy vector (Promega). Plasmids were purified using the Wizard Plus Minipreps DNA Purification System (Promega).

For construction of mosquito (host) phylogeny, we used a 987 bp region (excluding intron sequence) of Vg-C, a single copy yolk protein-encoding gene [30]. This region was amplified from *Ae. simpsoni *by nested PCR in our lab to add this species to the mosquito phylogeny. The following describes methods according to Isoe's work [30]. Degenerate primers were designed to amplify a 1.1 kb region that is specific for the Vg-C ortholog that includes the second intron. Primers Vg-C-specific forward (5'-(A/G)A(T/C)(A/G)TNAA(A/G)CA(T/C)CCNAA(A/G)G-3'), Vg-C-specific reverse (5'-TC(A/G)TT(T/C)TG(T/C)TT(A/G)TA(T/C)TG(A/G/T)CC-3'), and *Aedes *universal reverse (5'-C(A/G)T(A/G)CCA(A/G)CANTCNCCCAT-3') were used in nested PCR. The first PCR used the Vg-C-specific forward and reverse primers for 1 cycle at 94°C for 3 minutes, 32 cycles at 94°C for 1 minute, 50°C for 1.5 minute, and 1 extension cycle at 72°C for 10 minutes. The second PCR used the Vg-C-specific and *Aedes *universal reverse primers with the same conditions except that the annealing temperature was increased to 54°C. PCR products for *Ae. simpsoni *were cloned and sequenced as described above. Cloned inserts were sequenced in our laboratory using a GENE READIR DNA sequencer (LI-COR) with fluorescent-labeled T7 and m13r primers, or by DNA sequencing services (Amplicon Express and VBI-Blacksburg, VA). H_2_0 was used as a no-template negative control for PCR.

### Genome and sequence analysis

Genome analysis was performed on the contig version of the *Aedes aegypti *genome sequence, which consists of 36206 contigs comprising 1310.1 Mb, having 7.6 × coverage (Broad Institute). BLAST and other programs (TEpost, FromTEpost) developed in our lab [44] were used to extract and filter sequences from BLAST output. Genome contribution by *Juan-A *was estimated using RepeatMasker [28] using 70% identity cutoff with full-length *Juan-A *as query. The Wisconsin Package GCG version 10.2-UNIX (Genetics Computer Group) was used for analysis of cloned and sequenced PCR products. Alignments were produced with ClustalX 1.81 [48]. To obtain *dS/dN *values, substitution analysis was performed using the SNAP program on the web [49,50]. Only sequences that had intact sequence regions were used for substitution analysis. Mean values are calculated based on all pair-wise comparisons from that group.

### Phylogenetic inference

Phylogenetic inference was performed using MrBayes version 3.1.2 [51,52]. Sequences were aligned using ClustalX version 1.83 [53] using the following parameters: pair-wise alignment gap opening = 10, gap extension = 0.1; multiple alignment gap opening = 10, gap extension = 0.2. Nucleotide Vg-C sequence data (see above) were used for the host phylogeny. The Modeltest server (version 3.7) [54,55] was used to determine the best nucleotide evolutionary model (General Time Reversible (GTR) allowing for variable substitution rates among sites) according to an Aikaike Information Criteria (AIC) score. The model was implemented with MrBayes, running 150,000 generations, concluding with an average standard deviation of split frequencies below 0.01 (as suggested in the MrBayes manual), evidence of convergence of two independent tree searches.

Conceptually translated nucleotide sequences and sequences form Genbank (accessions) were used for non-LTR phylogeny. Sequences were aligned as described above. MrBayes was used to explore 9 fixed-rate amino acid evolutionary models, finding Jones [56] to have the highest score. Two hundred thousand generations were run resulting in an average standard deviation of split frequencies below 0.01. For all consensus trees displayed, clade credibility values are given at each node representing samplings of 1 of every 100^th ^generation, while discarding the first 25% of all generations (the "burnin" period). Another analysis performed for 1,000,000 generations produced the same tree topology. See additional files 1 and 2 for alignments used for phylogenetic inference.

### *Juan-A *copy number determination in the *Ae. aegypti *genome sequence

Different regions of the *Juan-A *sequence were used to determine *Juan *copy number in the *Ae. aegypti *genome by database search using BLAST (Figure 1, Table 1). The *Juan-A *3' UTR is approximately 240 bp. For copy number determination, we used 0.34 Kb of the 3' end as the query to be consistent with the use of the 0.34 Kb 5' UTR. Hits were counted which had sequence identities greater than or equal to 70%, 80%, 95%, 97%, 98%, or 99% compared to the query. In each case, a hit had to have at least 90% length of the query sequence. The full-length published *Juan-A *sequence is 4727 bp [24].

### Sequence identity comparisons

In Table 3, values shown for all species except *Ae. aegypti *are means plus one standard deviation from pair-wise comparisons of nucleotide sequences obtained by PCR. Only sequences from the same lineages are compared. Comparisons were made between sequences and the consensus derived from the number of sequences in column 3. *Ae. aegypti *sequences were obtained by database search using a query that spans the same sequence amplified by PCR (see Figure 1). The number 768 is higher than what is shown in row 1 of Table 1 because the query here is the segment used for PCR.

### Library screening

Amplified genomic libraries for *Ae. albopictus*, *Ae. polynesiensis*, *C. tarsalis*, and *C. quinquefasciatus *made using the Zap Express or Dash II kits (Stratagene) were screened using Digoxegenin-labeled (Roche Diagnostics) ssDNA probes generated from asymmetric PCR reactions. Two probes used for screening libraries of the *Aedes *or *Culex *genus were made from cloned PCR products amplified from *Ae. aegypti *and *C. tarsalis *using degenerate primers described above. The average insert size for the genomic libraries was 7 kb for *Aedes *and *Culex *libraries. Approximately 15,000 – 50,000 plaques were plated on NZY Agar plates and lifts were performed with nylon membranes (Osmonics). The membranes were blocked with prehybridization solution, containing 5 × SSC, 0.1% N-laurolylsarcosine, 0.02% SDS, and 2% nonfat milk for 2 hours at 55.0°C in a rotating hybridization incubator. Hybridization was performed with about 20 ng/ml of Digoxegenin-labeled probe in prehybridization solution for 6 hours to overnight at 55.0°C in a rotating hybridization incubator. Stringency washes were done using 0.5 × SSC, 0.1% SDS. Membranes were incubated with an anti-Digoxegenin antibody conjugated to alkaline phosphatase, and then developed with substrates BCIP and NBT for colorimetric detection. The copy number of *Juan *was calculated using known values of haploid genome size, average insert size of the library, and the ratio of positives to total number of plaques.

## Authors' contributions

ZT and JKB designed the study and drafted the manuscript. JKB performed sequence analysis and phylogenetic inference. All authors read and approved the final manuscript.

## Supplementary Material

Additional file 1 **alignment used for phylogenetic inference **(Figure 2A). Nexus format generated with Clustal Click here for file

Additional file 2 **alignment used for phylogenetic inference **(Figure 2B). Nexus format generated with Clustal Click here for file
